# 

**Published:** 1821-12-01

**Authors:** 


### BIBLIOGRAPHICAL RECORD;

OR

*Works received for Review, within the Quarter.*

1. A MANUAL for the STUDENT of ANATOMY; containing rules for displaying the structure of the body, so as to exhibit the elementary views of anatomy, and their application to pathology and surgery. By JOHN SHAW; being an outline of the demonstrations delivered by him to the students in the school of Great Windmill Street. One pocket volume, 8vo, pp. 342 of small type, with plates. London, September 1821.

---

2. Lectures on the Structure and Physiology of the Male Urinary and Genital Organs of the Human Body, and on the Nature and Treatment of their Diseases; delivered before the Royal College of Surgeons in London, in the summer of 1821. By JAMES WILSON, F R. S. Professor of Anatomy and Surgery to the College; Lecturer on Anatomy and Surgery at the Hunterian School in Great Windmill Street, &c. &c. One vol. 8vo, pp. 438, and three plates. London, September, 1821.

---

3. A Treatise on Cataract, intended to determine the operations required by different forms of that disease, on physiological principles. By PHILIP CHILWELL DE LA GARDE, Member of the Royal College of Surgeons, &c. One vol. 8vo, pp. 213. London, 1821.

4. Advice to the Young Mother, in the Management of herself and Infant. By a Member of the Royal College of Surgeons. Small 8vo, pp. 112. London, 1821.

*In common with the profession at large, we are adverse to medical works addressed exclusively to the popular reader. We seldom take the trouble to look into them, much less peruse them. The above little work was sent to us by a lady, whose literary lucubrations have been read and admired wherever the English language is understood. We consequently examined the publication, and we are constrained to say, that the work is too good for those to whom it is addressed; nor have we seen a single objectionable word or expression in it. We are totally unacquainted with the author's name.*

---

5. Rechearches Anatomico-Pathologiques sur l'Encéphale, et ses Dependances. *Lettre troisieme*, octavo. Paris, 1821

*Through M. Breschet.*

---

6, Revue Medicale, Historique et Philosophique. Juillet et Aout, 1821.

---

7. On the Nature, Symptoms, and Treatment of the different Species of Amaurosis, or Gutta Senera; illustrated by cases. By JOHN STEVENSON, Esq. Surgeon-Oculist and Aurist to His Royal Highness the Duke of York, &c. &c. One vol. 8vo, pp. 277. London, Oct. 1821.

---

8. On the Nature and History of the Marsh Poison. By WILLIAM FERGUSON, M.D. F.R.S. E. Inspector of Army Hospitals. (From the Transactions of the Royal Society of Edinburgh.) Quarto, pp. 29. Edinb. 1821.

---

9. The Influence of Tropical Climates on European Constitutions; being a Treatise on the principal diseases incidental to Europeans in the East and West Indies, Mediterranean, and Coast of Africa. By JAMES JOHNSON, M. D. Member of the Royal College of Physicians of London. One closely printed volume, 8vo, pp. 544, 3d edition. London, September 1821.

☞ *There are considerable additions to this edition; but they are chiefly for the benefit of those who are destined for the hotter climates of the earth. In this edition the subject of yellow fever occupies* 120 *pages of small type, and comprises a comprehensive review of the best modern works on the nature, causes, and treatment of that interesting disease. The East India division has been greatly enlarged by analytical reviews of several important works on the climate, and the publication is altogether carefully adapted as a manual for the tropical visitor.*

---

9. RECUEIL DE MEMOIRES DE CHIRURGIE, par le BARON LARREY, Chirurgien en Chief de l'Hopital de la Garde Royale, &c. One vol. 8vo, pp. 319, with numerous plates. Paris, 1821. *From the Author.*

☞ *We beg to return Baron Larrey many thanks for this interesting volume, and the polite letter which accompanied it. We shall take an early opportunity of making the English profession acquainted with this late work of the Baron's, knowing, as we do, the interest which the other writings of this experienced and able author have excited on this side of the Channel.*

11. Rapports et Consultation de Medecine Legale, recueillis et publiés. Par J. Ristelhueber, Docteur en Medecine, Medecin en Chef à l'Hopital Civil, &c. &c. a Strasbourg. Octavo, pp. 172. Paris, October 1821.

☞ *Transmitted by M. Breschet.*

---

12. Dissertatio Medica Inauguralis de Phthisêos Natura; Auctore Jacobo Black. Octavo, pp. 32. Glasguæ, 1821.

☞ *There are some ingenious speculations in Dr. Black's Thesis, respecting the different sanguineous capacities of the two circulations, pulmonic and aortic, and the consequent loss of equilibrium which occasionally takes place. We shall probably have an opportunity, on a future occasion, of making more particular allusion to this very respectable tentamen medicum.*

---

13. Philadelphia Journal of the Medical and Physical Sciences. No. 4, August 1821.

*This number contains*—1. *an able paper on yellow fever, by Dr. S. Jackson, in which the contagionists on both sides of the Atlantic are warmly handled—especially Sir Gilbert Blane in this country.* 2. *An historical account of syphilis, translated from the French.* 3. *On the generation of animal heat.* 4. *On cinchonine and kinine.* 5. *On inverted toe-nail.* 6. *On the application of atmospheric air to parts of the body not naturally exposed thereto.* 7. *On the devastation of the gums.* 8. *On the modus operandi of medicines, by Dr. Chapman.* 9. *On the tincture of poppy made from the entire plant, as a substitute for laudanum. The second department contains eight cases. The third contains five reviews. We shall notice several articles in the work, as soon as possible.*

---

14. Considerations sur la Fievre Jaune. Par le Baron Larrey, Chirurgien en Chef de l'Hopital de la Garde, &c, &c. Octavo, pp. 32. Paris, 1821.

---

15. A brief View of the Yellow Fever, as it appeared in Andalusia during the Epidemic of 1820: together with the mode of treatment adopted, and an account of the appearances *on dissection*. To which is prefixed, a short topographical sketch of the country. By Thomas O'Halloran, Esq. Octavo, pp. 171. London, November 1, 1821.

---

16. Journal of Popular Medicine, Nos. 8, 9, and 10. By Charles Haden, Surgeon to the Chelsea and Brompton Dispensary, &c.

---

17. A Treatise on the Diseases of the Chest, in which they are described according to their anatomical characters, and their diagnosis established on a new principle, by means of acoustic instruments; with plates. Translated from the French of R. T. H. Laennec, M. D. with a preface and notes by John Forbes, M. D. Physician to the Penzance Dispensary, Secretary of the Royal Geological Society of Cornwall, &c. &c. One vol. 8vo, pp. 431, and eight copper plates. London, 1821.

*In the 7th number of our Quarterly Series, for January* 1820, *we presented our readers with an analysis of the original work, of which Dr. Forbes has now given an excellent condensed translation, enriched with valuable notes, and illustrated by expressive plates. Time, and the general voice of the best judges in the profession, have confirmed what we said two years ago, namely, that Laennec's work was " one of the most valuable additions to our pathological knowledge of thoracic diseases which the present age has produced." We here again repeat our thorough conviction, " that those who neglect to possess themselves of the work either in the original or translation, inflict a deep wound on their best interests." We cannot, indeed, too strongly*

*exhort our brethren to study this invaluable repository of unwearied observation and indefatigable researches into the pathology of the most important division of the human body.*

---

18. Dissertatio Medica Inauguralis de Cordis Palpitatione. Auctore Carolo Locock, M. D. Edinburgh, 1821.

☞ *Dr. Locock dedicates this very respectable thesis to his friend and former preceptor, Mr. Brodie—"amico atque præceptori, publicis privatisque virtutibus illustri"—in grateful remembrance of the instruction and kindness received from that excellent and amiable surgeon. We were much pleased with the perusal of this thesis.*

---

19. Observations on those Diseases of Females which are attended by Discharges. Illustrated by copper plates of the Diseases. By Charles Mansfield Clarke, Member of the Royal College of Surgeons, London. Royal 8vo, pp. 243, five plates. London, Longman and Co. 1821.

☞ *This important work in our next.*

---

20. Notes on the Medical Topography of the Interior of Ceylon; and on the Health of the Troops employed in the Kandyan Provinces, during the Years 1815, 1816, 1817, 1818, 1819, and 1820: with brief Remarks on the prevailing Diseases. By Henry Marshall, Surgeon to the Forces. Octavo, pp. 200. London, 1821.

---

21. Miscellaneous works of the late Robert Willan, M.D. F.R. F.A.S. comprizing an Inquiry into the Antiquity of the Small-Pox, Measles, and Scarlet Fever, *now first published*—Reports on the Diseases in London, *a new edition*, and detached Papers on Medical Subjects, collected from various periodical publications. Edited by Ashby Smith, M.D. Licentiate of the Royal College of Physicians in London, &c. &c. One vol. 8vo, pp. 488. Cadel, London, 1821.

---

### TO CORRESPONDENTS.

W. S. has our thanks; but we cannot sully our pages by introducing any mention of the well-known Charlatan whom he exposes. The *Empirical Journal* to which he alludes needs no comments *in* the profession—and *out* of the profession our Review has but a very limited circulation.

In respect to the *calumniating tribe*, we beg to refer W. S. to the 113th page of the 9th edition of "Lacon; or, many things in few words," where he will find the following passage:—

"There are two modes of establishing our reputation;—to be praised by honest men, and to be abused by rogues. It is best, however, to secure the *former*, because it will be invariably accompanied by the *latter*. His calumniation is not only the greatest benefit a rogue can confer upon us, but it is also the only service that he will perform for nothing."

Mr. Mogridge's communication respecting the mode of managing fractured patella, came to hand, but as we cannot break our plan by introducing original communications, we have transmitted the paper to one of our cotemporaries for publication. After its appearance in the proper place, it will be noticed in our Quarterly Periscope.

It is quite unnecessary for us to insert Argus's "report" of a pretended medical society, and the issue of a Journal from such a source. It is only another "MEDICAL BOARD"—and "by their fruits ye shall know them."

The excellent discourse on the evidences of revealed religion, by the Rev. Mr. Channing, transmitted to us from Boston, in America, in consequence of our review of Pring on the "Laws of Organic Life," has been received. The request that it may be transmitted to Mr. Pring, after perusing it, has been complied with.

### MISCELLANIES.

### MEDICAL PUPIL.

A physician attached to several public institutions is desirous of accommodating a respectable house pupil. The terms and peculiar advantages of the residence here proposed may be known by reference to Dr. James Johnson.

### *Additional Subscribers since last Quarter.*

A.

Andrew, Mr. Surgeon, Fetter Lane

B.

Barbadoes Military Medical Society

Berth, Mr. James, Surgeon, Halifax, Yorkshire

Birdsall, William, Esq. Surgeon, Syston, Leicestershire

Black, Dr. James, Bolton, Lancashire

Black, Mr. Surgeon, Back Road Islington.

Bonsfield, Dr. Spilsby.

Brewster, James, Esq. Tollinghunt, Darcy

Buckley, Mr. Surgeon, Thursk

Bull, Cristopher, M. D. Cork

C.

Corfu Garrison Medical Library

Coley, Dr. R. W. Resident Physician, Cheltenham

Cork Institution

Christie, Robert, Esq. Hon. East India Company's Service

Crisp, B. J. Esq. Surgeon, Brixworth, Northamptonshire

Cooper, Mr. Charles, Surgeon, Upper Tooting

Carruthers, Mr. Surg, Romford

Christie, Mr. Spencer Street, Northampton Square

Cooke, W. Esq. Surg. Dover

D.

Drake, Dr. Hadleigh Suffolk

Dill, Dr. Marquis, Newton Limavady, Ireland

Dickinson, Mr. Josiah, Croxton, near Chorley, Lancashire

Davies, Dr. W. A. Tenby

Duncan, Mr. Surgeon, Wrotham

E.

Edwards, Henry, Esq. Bartholomew's Hospital

F.

Filkin, Dr. Thomas

G.

Griffith, Dr. Philadelphia
Gedikė, Dr. C. E. Berlin
Grady, Mr. C, (place not stated)

H.

Hey, Mr. Surgeon, Kenninghall

I. J.

Jackson, Robert, M. D. Stockton on Tees
Jones, Mr. Henry, Surgeon, Welsh-Pool
Illingworth, Mr. Surgeon, Bradford, Yorkshire
Jice, Mr. Surgeon, Ware
Ivory, Mr. Surg. Nassau Street

K.

Keddel, Mr. Surgeon, Deptford

L.

Laird, Dr. Physician to Guy's Hospital, Bloomsbury Square

M.

M'Manus, Mr. R. Paris
Maclean, Sir L. Physician, Sudbury, Suffolk
Maclean, Allan, M. B. one of the Physicians to the Essex and Colchester Hospital.
Mansel, Thomas, Esq. Surgeon, Pembroke
Mann, George, Esq. Surg. Cork
Macmichael, Dr. Fellow of the Royal College of Physicians Albany, Piccadilly
Medical Library, General Infirmary, Northampton
Mackintosh, Dr. R. D. (late Physician to the Essex and Colchester Hosp.) Queen Square, Bloomsbury
M'Crea, Dr. James, Royal Navy
Murray, Mr. Surgeon, Frome

N.

Newman, M. Esq. Surgeon, Mere, Wilts
Nash, Mr. James, Surgeon, Hartleberry, Worcestershire

O.

Owen, W. Esq. Surg. Glandrilay, near Llanidloes, Wales
O'Donel, Dr. 4th Dragoons, Bombay

P.

Peterborough Dispensary, Northamptonshire

R.

Rann, Dr. Benborough, Oxfordshire
Round, Mr. Cheque Office, Bank
Ramadge, Francis, H. Member of the Royal College of Physicians, and Physician to the Infirmary for the cure of Asthma, Consumption, &c. Union St. Bishopsgate
Ridge, Mr. Benjamin, Surgeon, &c. Bridge Road, Lambeth
Robinson, Dr. Princes Street, Stamford Street
Rowe, Matthew, Esq. Surgeon, Burton Crescent

S.

Surrage, T. L. Esq. Surgeon, Wincanton, Somersetshire
Scripp, Mr. Surgeon, Welsh Pool
Shillitoe, Richard, Member of the Royal College of Surgeons in London, Hartford
Spurgin, Mr. Thomas
Seymour, Mr. J. G. Bishops Waltham
Sutherland, J. A.M. Surgeon, Southwold, Suffolk

T.

Terry, Henry, Esq. Surgeon, Northampton

W.

Wake, Dr. York
Walker, Dr. Physician to the British Embassy, St. Petersburgh
Woodall, Mr. Robert, Member of the Worshipful Society of Apothecaries, Manchester
Welsh, Mr. W. Surgeon, Plymouth Dock

Y.

Young, Jas, Esq. Surg. Wells

---

*No.* VIII. *will be published on the* 1*st of March*, 1822.

